# 218. A 5-Year Retrospective Study of Actinomyces Odontolyticus Bacteraemia

**DOI:** 10.1093/ofid/ofab466.420

**Published:** 2021-12-04

**Authors:** Maisa Ali, Almurtada Razok, Hisham Ziglam

**Affiliations:** HMC, Doha, Ad Dawhah, Qatar

## Abstract

**Background:**

Actinomyces species are Gram positive anaerobic, non-sporulating, non-acid fast, non-motile, irregularly staining bacterium. It is associated with a wide range of infections including; dental caries, abscesses, intraabdominal and bloodstream infections. A. odontolyticus normally a commensal organism found in the mouth, was first isolated from dental caries in 1958. The incidence of Actinomyces odontolyticus bacteremia is less common.

**Methods:**

We are reporting 15 cases of isolated A. odontolyticus blood stream infection at HMC, State of Qatar from 1/1/2016 to 1/11/2020. We aim to describe their clinical characteristic, risk factors and treatment outcome.

**Results:**

Our patients with bacteraemia fall into one of two groups. The first group consists of paediatric patients with unremarkable co-morbidities. The second group includes older adults, often with co-morbidities that pre-dispose to infection, such as diabetes mellitus or hypertension. Fever was the main presenting sign and symptom in 12 patients (80%). Nine of the patients were females (60%). 13 patients (86%) received antibiotics. Maximum duration of antibiotics was 60 days and minimum duration was three days. The infectious disease team was consulted for six patients (40%). One patient died while the other 14 recovered uneventfully with a case fatality rate of 6.6%.

Reported cases of Actinomyces odontolyticus bacteraemia in Hamad Medical Corporation between 1/1/2016 to 1/11/2020

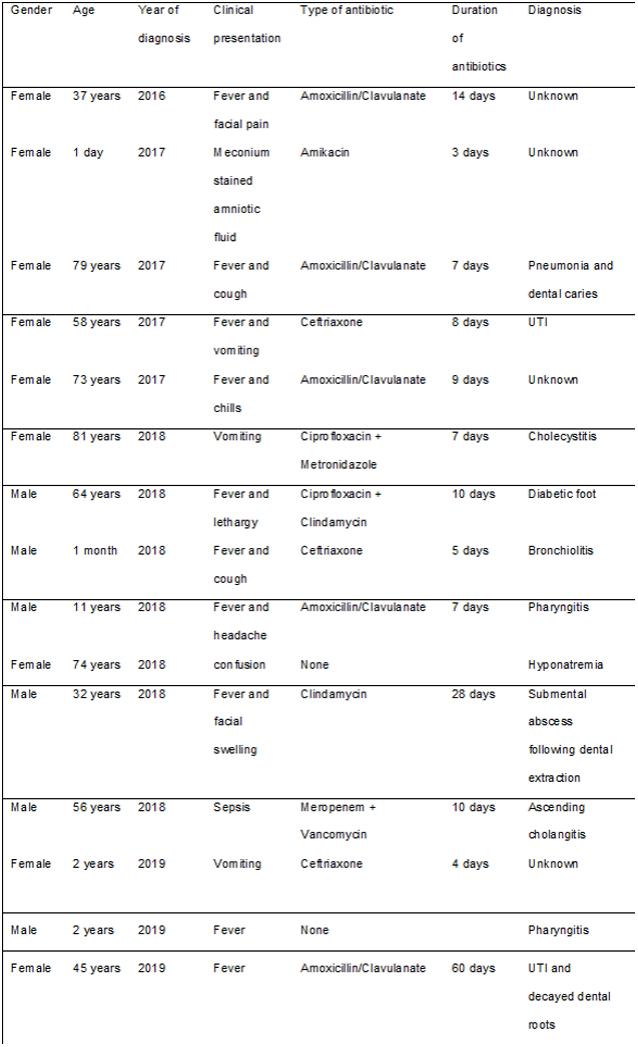

Minimal inhibitory concentrations (MICs) of selected antibiotics against A. odontolyticus, including interpretations and breakpoints, as reported by the AMRHAI reference unit, PHE Colindale

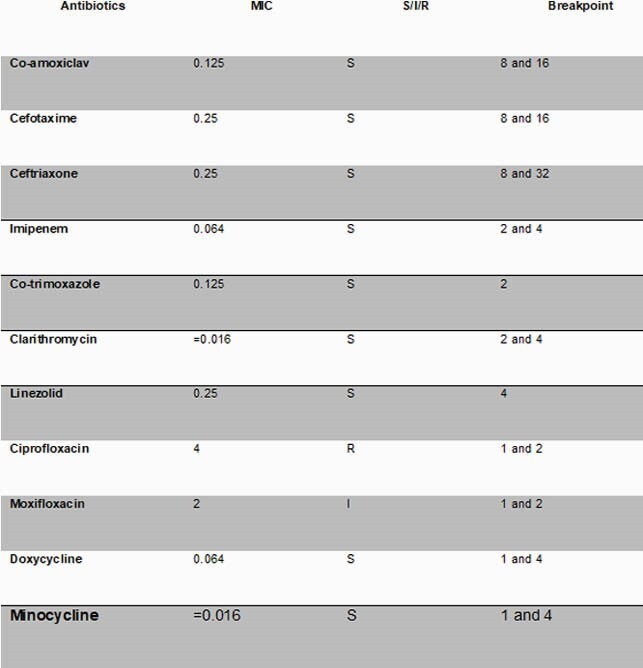

**Conclusion:**

Clinicians of all specialties need to be aware of the rising number of reports of Actinomyces species bacteraemia due to widespread availability of molecular identification techniques, including MALTI-TOF. 3 Furthermore, more studies are needed to determine guidelines for treating these resilient microbes

**Disclosures:**

**All Authors**: No reported disclosures

